# Trials of Life

**Published:** 1880-10

**Authors:** 


**TRIALS OF LIFE.**—We start upon life's journey full of hope, full of gladness, and full of joyous ambition, confident in our own strength and in the support of friends and kindred stationed round about us, on whom we lean with great satisfaction; but as years pass on, one of the outposts, the supports, falls; and then another and another, each succeeding year, leaving one or more the less. For a while we scarcely miss the acquaintances and friends of our childhood, for we have so many; but as time rolls on, the number becomes so small that each additional loss makes a greater void. Father, mother, brothers, sisters, our oldest neighbors, all gone; the minister of our youth has grown gray before us—he, too, has passed away; and beyond a schoolmate here and another there, nothing is left to connect us with the times and the home of childhood, and such a feeling of desolation comes over us, that we are ready to sink in perfect helplessness and despair. To the old who may chance to read these lines, the suggestion is made, which, if wisely heeded, may save the body from sinking under the whelming load, and it is this, He who made us, is the Father of us all; and the dispensations of this life are designed to prepare us the more certainly for a beatific existence beyond the grave, and to enable us to make the transition with the least violence, and at the same time to train us to those habitudes of heart which will the more elevate us in the world beyond, he arranges that we shall learn to lean less on ourselves, less on others, and more on Himself, as a weary man leans on a staff; and the sooner we begin to learn thus to lean, the happier we shall be in time, and the more ready shall we find ourselves to take up the returnless journey without a murmur and without a sigh.

### WANTED BADLY.

A good many sentimental tears have been shed over that phantasy of an inspirited brain, "The song of a shirt." There is no doubt the writer had a glass of brandy, and a pipe beside him, when he wrote all that rigmarole about "stitch, stitch, stitch." There is nothing like "leather," but hard facts. Some time ago, a friend wanted a dozen shirts made, and asked our aid in finding a seamstress; attracted by a sign, a young woman presented herself; she was asked to name her price for making one; and was told that if the work was well done she could have the remainder at her own price. The article was dilatorily made, sent home and paid for; the bosom buttons came off before the man was dressed; on examination, it was found that they were attached by a single thread, and even that, loosely. Who ever purchased a ready made garment that did not want repairing after a week's use, either as to buttons or rips. We feelingly know that a really good dressmaker commands two dollars a day, coming at eight o'clock and leaving at six, wanting two very hearty meals, with tea at both, two or three cups of it, of the strongest kind, costing one dollar and a half a pound; and to get this same woman, even for a day, is sometimes the work of a month, that is, she generally is engaged that long before hand; to be sure she understands her business, and does it well. A common sewer demands a dollar a day and board; but seldom can be had without a fortnight's notice; there are hundreds of families in New York city to day, who would gladly pay high prices for a person who could sew well. There are five thousand families in New York city who would cheerfully give from fifteen to twenty dollars a month for a cleanly, capable, honest and economical cook, who had no relations to feed; who had no visitors, and who retired at ten o'clock. There are five thousand places for house girls who are fully competent to the duties of their department. It is scarcely possible to go into any mechanical office or shop where work is well done, and get it promptly done, because such men have more work than they can do. It is only the incompetent who fail to do well in New York.

